# Comparing the effects of contact duration on cow and calf performance beyond separation - a prospective cohort study

**DOI:** 10.1186/s13028-024-00741-1

**Published:** 2024-05-22

**Authors:** Johanne Sørby, Ingrid Hunter Holmøy, Ane Nødtvedt, Sabine Ferneborg, Julie Føske Johnsen

**Affiliations:** 1 Department of Animal and Aquacultural Sciences, Faculty of Biosciences, Norwegian University of Life Sciences, Oluf Thesens vei 6, Ås, 1433 Norway; 2 Department of Production Animal Clinical Sciences, Faculty of Veterinary Medicine, Norwegian University of Life Sciences, Elizabeth Stephansens vei 15, Ås, 1433 Norway; 3 Section for Terrestrial Animal Health and Welfare, Norwegian Veterinary Institute Elizabeth, Stephansens vei 1, Ås, 1433 Norway

**Keywords:** Calf growth, Dam-rearing, Milk yield, Post weaning

## Abstract

**Background:**

Consumers, the industry, and many farmers have shown increased interest in alternative management systems that allow for cow-calf contact (CCC) and this topic has become an important focus of research for a sustainable dairy industry. Among the many knowledge gaps still existing in this novel research field, there is a need for further research to investigate effects of CCC beyond the nursing period (i.e., after cow-calf separation). Moreover, multi-herd observational studies are scarce. Therefore, the aim of this study was to investigate the within-herd effect of CCC on machine milk yield and calf growth beyond separation. In this prospective cohort study, we studied all cows and their calves (Norwegian Red breed) born between September 1st 2021 and January 31th 2022 at three Norwegian dairy farms, investigating the impact of CCC on machine milk yield and calf average daily gain (ADG) after separation of the cow-calf pair. The follow-up period was 305 d for cows and six months for calves. Machine milk yield was automatically registered at each milking and calf heart girth was measured once a month. As various contact durations emerged, they were categorized into three groups: no contact (NC 0–3 d), short duration (4–30 d) and long duration (> 30 d). Data were analyzed with linear mixed models with the individual animal as the unit of interest. For cows, days in milk (DIM) from date of separation up to 305 DIM were included in the analysis as a continuous variable. For calves, age up to 195 d was used in the statistical analysis.

**Results:**

We found no differences in machine milk yield after separation across the different contact groups: cows with no contact (*n* = 28), short duration (*n* = 51) and long duration (*n* = 27) of contact, all exhibited comparable milk yields from the time of separation until the end of lactation. Furthermore, in the case of calves, no disparities in average daily gain (ADG) were identified for any of the contact groups: calves with no contact (*n* = 39), short duration (*n* = 61) and long duration (*n* = 38) of contact, displayed comparable growth during the first six months of life.

**Conclusions:**

Our findings indicate no negative effects of CCC on machine milk yield after separation, nor any sustained effects on calf growth under the conditions of this study. More multi-herd observational studies conducted on-farm is needed to expand the understanding of effects of CCC on cow and calf performance after separation.

**Supplementary Information:**

The online version contains supplementary material available at 10.1186/s13028-024-00741-1.

## Background

On modern dairy farms, cows are typically separated from their calves within 24 h of birth. Calves are then fed milk either through buckets or bottles until they are weaned sometime between eight and 12 weeks of life [1], also referred to as artificial calf rearing [2]. This practice differs from natural conditions, where cows start leaving their calves in groups when the calves are about two weeks old, while they graze nearby [3]. The cow and calf remain together until the calf is gradually weaned at around 7 to 10 months of age [4]. The practice of early separation of cow and calf is connected to tradition, economic considerations, and concerns for the health of both the cow and calf [5, 6]. However, in recent years, the practice of early separation has faced criticism in discussions about dairy farming within society [7, 8]. Consumers, the industry, and many farmers [6, 9] have shown increased interest in alternative management systems that allow for cow-calf contact (CCC) [2]. This topic has become an important focus of research for a sustainable dairy industry [10, 11]. In such systems, cows and their calves can stay together for an extended period, enabling them to engage in natural behaviors such as allogrooming, suckling, play and care-taking [12]. However, many knowledge gaps still exist in the novel research field of CCC.

In some CCC systems, the cows’ milk is partly suckled by the calf (usually not quantified) and partly harvested at milking (machine milk yield) [2]. Multiple studies have reported that allowing cows to nurse their calves decreases the volume of machine milk yield during the nursing period [13–15], but there is no consistent evidence of a reduced machine milk yield beyond separation [13]. This was supported in a recent study where it was found that machine milk yield of whole-day (24 h/d) [2] contact cows seemed to recover to some extent once the calves were weaned [14]. Additionally, in a recent controlled CCC study comparing effects of gradual debonding, we observed that cows react to reduction in contact with increased machine milk yield [16]. Contrary to this, there are studies who have found that machine milk yield from whole-day contact cows remained lower compared to part-time (half-day or multiple short times/d) [2] and control cows even after the calf separation [17, 18]. These inconclusive findings emphasize the need for further research to investigate effects of CCC on machine milk yield beyond the nursing period (i.e., after cow-calf separation).

Multiple previous studies have demonstrated that dam-reared calves experience significantly higher weight gain during suckling compared to artificially reared calves subjected to milk restriction (milk fed to 10% of BW) during the milk feeding period [13, 19]. However, when weaning off milk and separation from the dam occur simultaneously, CCC calves tend to suffer from a drop in growth (growth check) [13, 20, 21]. Studies investigating the weight gain of dam-reared calves beyond separation have yielded inconsistent results. While many studies have reported sustained growth benefits for several weeks or months following separation [13, 22], other studies have reported lower ADG of whole-day contact calves compared to control calves two [20] and six [21] weeks after separation. Similarly, the growth advantage of whole-day contact calves during suckling was no longer detectable at six months of age in a recent study [14]. More knowledge is needed on potentially prolonged effects of CCC on calf growth.

These inconsistent previous findings highlight the complexity of calf growth and milk production dynamics in CCC systems and the need for further research on potentially prolonged effects of having cows and calves together. Most previous studies on this topic have primarily been conducted in single-site research facilities under controlled experimental settings. Multi-herd, observational studies from the “real-world” are needed to expand our understanding on the causative effects of CCC on cow and calf performance [23]. Therefore, the aim of this study was to investigate the within-herd effect of CCC on machine milk yield and calf growth beyond separation.

By looking at the period from separation until the end of lactation, we hypothesized that daily machine milk yield would not be affected by CCC, while calf growth would be positively affected by CCC the first six months of life.

## Methods

### The Norwegian context

Norwegian dairy farming is small-scale by most European standards, with an average farm size of 30.9 cows per herd in 2022 and with a total of 6,925 registered dairy herds [24].

Norwegian agriculture is multifunctional and technology-driven, where over 47% of the milk output is derived from dairy farms with automatic milking systems (AMS) and the percentage is increasing [25]. In the year 2022, the productivity of each cow reached an average of 8,191 kg of milk. Notably, 91.3% of the dairy cow population in Norway comprised the Norwegian Red (NRF) breed, a dual-purpose breed (bred to produce both meat and milk) [24].

### Recruitment of herds

**Fig. 1:**
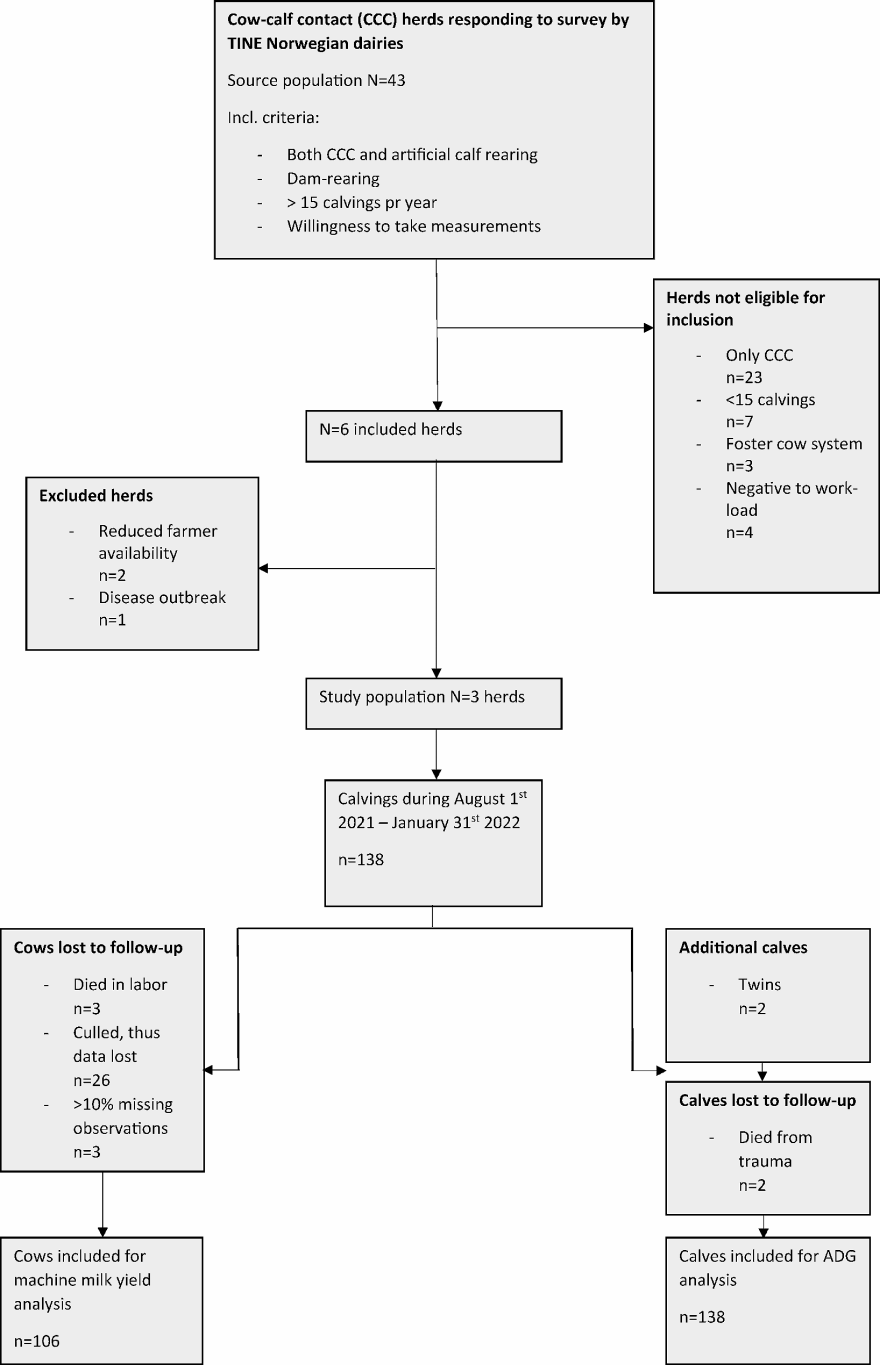
Flowchart Flowchart of eligible and analyzed herds in a prospective cohort study comparing different cow-calf contact durations

In early 2020, TINE Norwegian dairies sent out a survey to all dairy herds practicing CCC in Norway. The survey was distributed in two main channels: TINE dairies’ advisory services sent the survey to farmers known to be practicing CCC and a link to the survey was published on a Facebook group “Forum for CCC-interested farmers in Norway” with 1500 members (as of September 2021). The list of respondents from this survey made the base for recruitment to our study. The aim of our study was to compare the effects of CCC within herds, which required engaging farmers who practiced both CCC and artificial calf rearing. Additional inclusion criteria and an overview of eligible and analyzed herds are shown in Fig. 1.

### Study design

In this prospective cohort study, we studied all cows and their calves born between September 1st 2021 and January 31st 2022 in three Norwegian dairy farms. For one of the included herds, inclusion was initiated in August 2021 to coincide with the time of peak calving. Each farmer decided when to separate cow and calf and thus the duration of contact for each pair. The reason for separation was noted for each pair, but all three farmers based these decisions on available space and the cows’ maternal behavior. The follow-up period was 305 d for the cows and six months for calves. All procedures were in accordance with the regulations controlling experiments/procedures in live animals in Norway, and the study complied with the policies relating to animal ethics.

### Data collection

During July 2021, herds eligible for inclusion were visited by the first author, and a questionnaire was completed in collaboration with each farmer. The questionnaire addressed data on herd size, the farmer’s method of CCC, milk allowance for artificially reared calves, and other relevant information. The questionnaire can be found in Additional file 1. Each farmer also got written instructions on how to measure calf heart girth by using tapes converting measurements into live weight in kg [26], and received forms for registrations (details below, form can be found in Additional file 2).

Upon calving, the farmer recorded individual animal data related to the exposure (CCC): date and time of calving and cow-calf separation. In addition, the farmer recorded other explanatory variables such as, cow and calf id, cow parity, any calving difficulties, calf sex, crossbreed of cow or calf (if any) and whether the cow had prior experience caring for a calf. Furthermore, the farmer registered date of loss to follow up (culling etc.).

The outcome variable for cow performance was individual machine milk yield. All cows in the study were milked by an AMS, and the machine milk yield of each individual cow and DIM was automatically registered using Delpro software (DeLaval International AB, Tumba, Sweden) or Horizon software (Lely Astronaut A4, Maassluis, the Netherlands) at every milking. Data was remotely downloaded by the first author every third month throughout the lactation.

The outcome variable for calf performance was individual ADG (kg). Farmers measured heart girth of each calf at birth and then the first week of each month for the six first months after calving, independent of age. Measurements were recorded in a form. Monthly reminders and encouragement were sent to each farmer during the follow-up period.

### Statistical methods

Basic data handling was performed in Excel (version Office 2016, Microsoft). All statistical analyses were performed using Stata (Stata SE/16, Stata Corp., College Station, TX, USA). The experimental units in this study were individual cows and calves.

The main predictor (i.e., exposure of interest), was CCC (days) for each pair. Based on the dates of separation, we assessed the distribution of CCC within and between herds using histograms (results not shown). We calculated mean values and spread of CCC for each herd. We observed a wide range of contact among pairs, which ranged from 0 to 67 d (18.4 ± 20.5, mean ± SD). For better visualization of data and to reflect the variation in CCC between herds, we categorized the observations based on the variation in contact into three groups based on histogram evaluation, hereafter referred to as contact durations. Specifically, we refer to the artificially reared calves as the “no contact” (NC) group, being calves with 0–3 d of contact [27]. Further, we classified calves that had a contact duration of 4–30 d as the “short duration” group, and those with more than 30 d of contact were referred to as the “long duration” group. Cow parity was classified as either primiparous or multiparous. Cow and calf breed was classified as NRF or “others” (i.e., STN, Sidet Trønder og Nordlandsfe, traditional Norwegian breed or Aberdeen Angus). The low variation in farmers’ records of calving difficulties prompted us to classify it as “yes” or “no”. Likewise, whether the cow had previously cared for a calf was also categorized as either “yes” or “no”. Calf age was calculated based on date (weighing date relative to calving date).

### Outcome variables

Cow daily machine milk yield was calculated per 24 h using moving averages over three consecutive days, due to high day-to-day variability. We assessed the distribution of machine milk yield among the different cow-calf contact groups using histograms (results not shown). Across all individual animal explanatory variables, we calculated descriptive statistics (mean and SD) for machine milk yield. For description, mean daily machine milk yield from 0 to 305 DIM was visualized graphically using individual cow line graphs across contact duration and parity. Machine milk yield from primiparous NC cows is not presented as a separate group as this group consisted of only four cows.

For description, we calculated mean and 95% CI live birth weight of calves across month (d < 30 = month 1, d > 30 < 60 = month 2) and cow-calf contact groups. To calculate ADG, each calf’s weight increment since last measurement was divided by the number of days since last measurement. We assessed the distribution of ADG among the different cow-calf contact durations using histograms (results not shown). Across all individual animal explanatory variables, we calculated descriptive statistics (mean and SD) for ADG.

### Statistical analyses

Initially, mixed univariable linear regression models were built to assess how the outcome, mean daily cow machine milk yield, was associated with the exposure (no contact, short duration, long duration) and individual cow explanatory variables (parity, calving difficulties, breed, calf age, calf sex, experience with caring for a calf, DIM). There were different milking permission strategies both between and within herds (range 1–18/d), consequently, milking frequency was not incorporated into the analysis in our study. Variables that did not explain a considerable variation in machine milk yield (*P* > 0.1 in univariable analysis), were not considered further.

A multivariable linear mixed model was then built to analyze mean daily cow machine milk yield. Post estimation, a Wald test was used to test the hypothesis that the categorical variable contact duration was associated with the outcome. Our study focused on the period from separation until the end of lactation, and therefore DIM before separation were not included in the analysis. Cows with less than 30 observations after separation were removed from the dataset [23]. The lactation curve was modeled by including both DIM and DIM transformed to a natural logarithmic scale (lnDIM) [28].

Model selection followed a stepwise regression process starting with a forward selection with all explanatory variables and biologically plausible interactions (contact duration×parity) followed by a backwards stepwise removal of each variable based on significance [23]. To account for the hierarchical structure of the data and lack of independence between observations from animals within a herd and the same individual over time and, herd and animal id were included in the model as random effects. A first-order autoregressive covariance structure was applied to account for the observed temporal correlation. The effect of breed was not included in further analysis, as there were too few and unequal observations across herds. In addition, calf breed was confounded with parity of the dam. The best fitted model was chosen after comparisons of Akaike information criterion (AIC).

For calf performance, we followed the same model building process as for cow machine milk yield. The explanatory variables in the maximum model were: cow-calf contact duration, dam parity, calf sex, breed and calving difficulties.

Significant differences were declared at *P* ≤ 0.05. Data is presented as marginal means ± SE unless otherwise specified.

## Results

### Descriptive statistics

The dataset included a total of 106 cows, on average 241 (range 30–306) observations per cow. There were 36 primiparous and 70 multiparous cows, with an average lactation number of 2.4 ± 1.3 (mean ± SD). A total of 138 calves were included, on average 4.8 (range 2–9) observations per calf. Herd characteristics are summarized in Table 1. Notably, two herds aligned with the national average herd size, registering 40 cows per herd compared to the national average of 30.9 cows per herd [24]. However, these herds exhibited a slightly lower annual milk yield than the national average, ranging from 7,000 to 7,500 kg compared to the average of 8,191 kg [24]. In contrast, the third herd surpassed both the national average herd size and average annual milk yield, with 130 cows per herd and an average of 8,500 kg of milk.

**Table 1:** Herd information from a prospective cohort study with different cow-calf contact durations

		Herd		
		1	2	3
General herd information	Herd size (nr. of lactating cows)	40	40	130
	Type of farming	Conventional	Conventional	Conventional
	Animal housing	Freestall	Freestall	Freestall
	Milking system	AMS	AMS	AMS
	Average annual milk yield (kg Energy-corrected milk)^1^	7000	7500	8500
	CCC housing	Freestall	Freestall	Individual pens
	Separation strategy	Abrupt^2^	Abrupt	Abrupt
	Milk allowance for no-contact calves	2–3 L 2x/d	Ad lib	Ad lib. 4 wks, then 2 L 2x/d
Study population and loss to follow-up	Included cow-calf pairs, n.	39	27	72
	Cows lost to follow-up^3^	19	2	11
	Calves lost to follow-up	0	2	0
Cow-calf contact duration (mean ± SD)^4^		28.4 ± 17.52	36.0 ± 28.00	8.0 ± 7.13

^1^self-reported

^2^ [2]

^3^see Flowchart in Fig. 1

^4^includes no-contact group

The NC group comprised 28 cows and 39 calves, the short duration group 51 cows and 61 calves, and the long duration group 27 cows and 38 calves.

### Cow performance

**Fig. 2:**
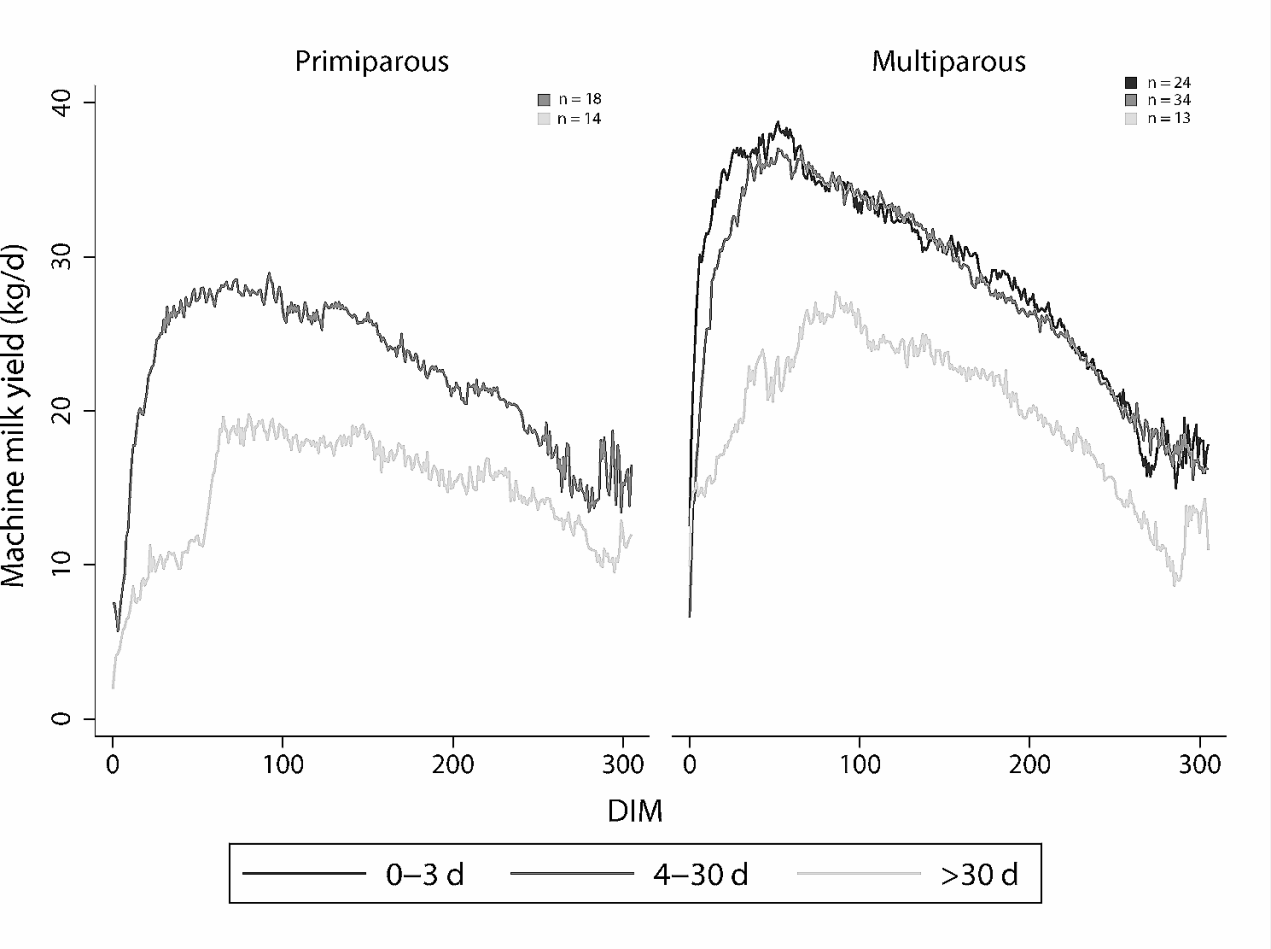
Mean machine milk yield Mean machine milk yield (kg/d) from primiparous and multiparous cows with different cow-calf contact duration (0–3 d, 4–30 d, > 30 d), in a prospective cohort study at three herds

Overall, average machine milk yield was 25.6 ± 8.58 kg/d (mean ± SD), with many missing observations on machine milk yield during the period of nursing as many cows were bucked-milked resulting in unregistered quantities. Figure 2 shows machine milk yield from 0 to 305 DIM across contact duration and parity. The distribution of machine milk yield across the explanatory variables tested in univariable analysis can be found in Table 2.

**Table 2:** Individual cow explanatory variables tested in univariable analysis from a prospective cohort cow-calf contact study

		Machine milk yield (kg/d)		
Variable	Class	*n*	Mean	SD
Cow-calf contact duration	No contact (0–3 d)	28	27.9	8.65
	Short duration (4–30 d)	51	26.5	8.26
	Long duration (> 30 d)	27	17.6	7.28
Parity	Primiparous	36	19.4	7.44
	Multiparous	70	27.0	8.78
Cow breed	NRF^1^	104	24.7	9.07
	Crossbreed^2^	2	21.1	10.05
Calf breed	NRF	94	25.6	8.72
	Crossbreed^3^	12	15.9	7.87
Calving difficulties	Yes		27.5	8.78
	No		24.4	9.09
Previous experience with CCC	Yes	56	21.3	8.53
	No	50	27.3	8.67

^1^Norwegian Red

^2^Sidet Trønder og Nordlandsfe (STN, traditional Norwegian breed)

^3^STN and Aberdeen Angus

### Calf performance

**Fig. 3:**
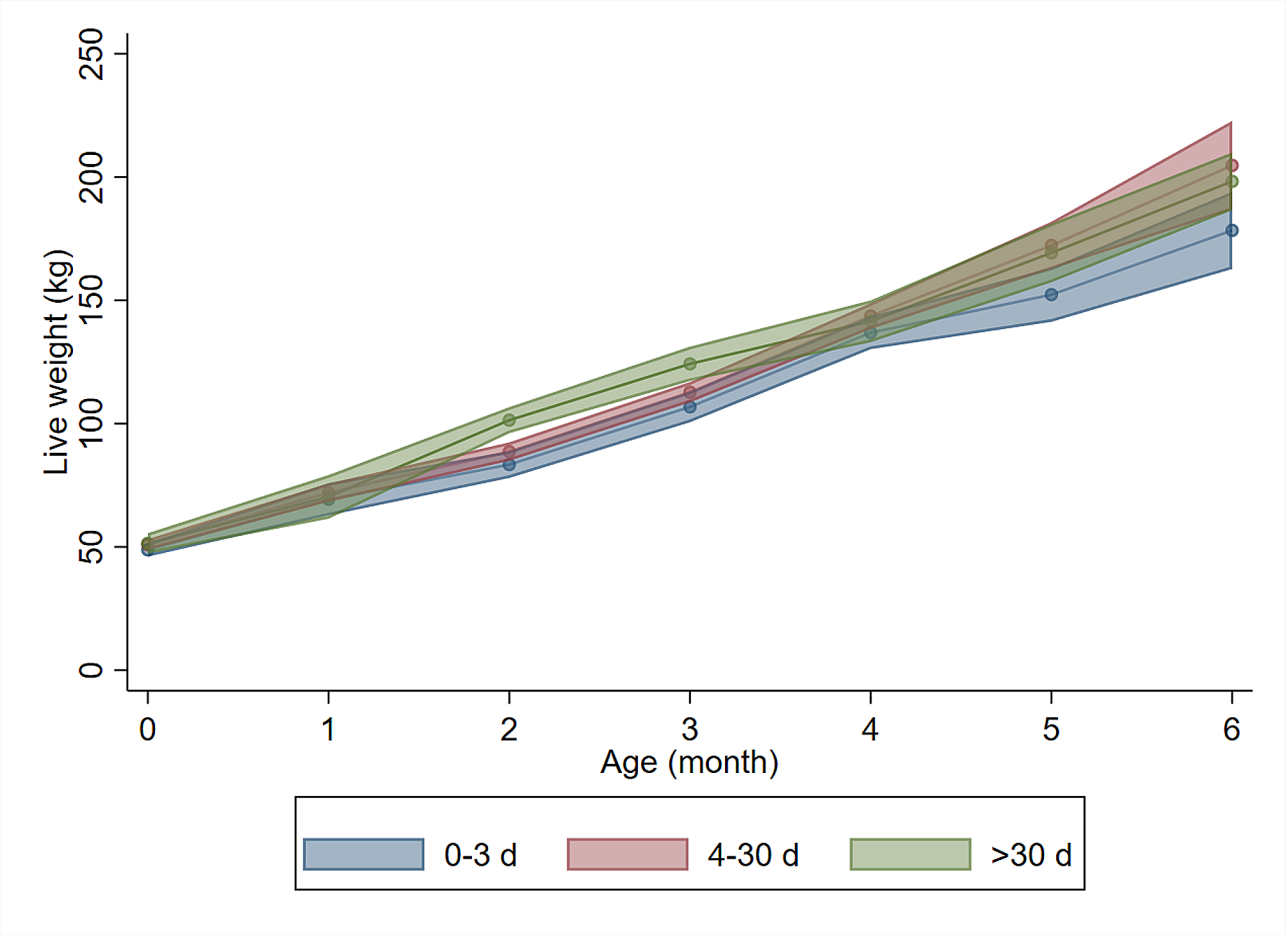
Mean monthly live weights Mean monthly live weights (kg) of calves with different cow-calf contact duration (0–3 d blue, 4–30 d red, > 30 d green), in a prospective cohort study at three herds. Shaded areas indicate upper and lower 95% confidence intervals

There were 64 heifer calves and 74 bull calves in the study, with an overall average ADG of 0.96 ± 0.60 kg/d (mean ± SD). Figure 3 shows calves’ absolute body weight during the first six months of life. One herd practiced abrupt separation from the dam and simultaneous weaning, while the other two herds practiced abrupt separation with a transition to artificial milk rearing. Calf mortality during the study was low, two calves (1.4%) died from trauma but none due to illness. The distribution of ADG across the explanatory variables tested in univariable analysis can be found in Table 3.

**Table 3:** Individual calf explanatory variables tested in univariable analysis from a prospective cohort cow-calf contact study

		Calf ADG (kg/d)		
Variable	Class	*n*	Mean	SD
Cow-calf contact duration	No contact (0–3 d)	39	0.9	0.52
	Short duration (4–30 d)	61	1.0	0.64
	Long duration (> 30 d)	38	1.0	0.60
Parity	Primiparous	51	0.9	0.51
	Multiparous	87	1.0	0.64
Calf sex	Heifer calves	64	0.9	0.52
	Bull calves	74	1.0	0.42
Cow breed	NRF^1^	136	1.0	0.60
	Crossbreed^2^	2	1.0	0.70
Calf breed	NRF	124	1.0	0.60
	Crossbreed^3^	14	0.9	0.53
Calving difficulties	Yes	11	1.0	0.45
	No	127	1.0	0.59

^1^Norwegian Red

^2^Sidet Trønder og Nordlandsfe (STN, traditional Norwegian breed)

^3^STN and Aberdeen Angus

### Statistical analyses

#### Cow machine milk yield

The following explanatory variables did not explain a considerable variation in machine milk yield (*P* > 0.1 in univariable analysis): calving difficulties, previous experience with caring for a calf, and calf age as a categorical variable. The final model for machine milk yield consisted of the explanatory variables contact duration, parity, DIM and lnDIM, in addition to the random effect of cow and herd. DIM from date of separation up to 305 DIM were included in the statistical analysis.

The Wald test revealed no statistically significant association between contact duration and daily machine milk yield from separation throughout lactation (*P* = 0.084, Table 4). Multiparous cows showed a significantly higher machine milk yield than primiparous cows, and no interaction between parity and contact duration was found. The random effect of herd explained 6.6% of the residual variation and cow id 63% of the residual variation.

**Table 4:** Results from linear mixed model on mean daily cow machine milk yield (kg/d) after separation

		Coefficient	SE	95% lower CI	95% higher CI	*P*
Intercept		25.47	1.86	21.829	29.121	< 0.001
Contact duration	No contact (0–3 d)	-				
	Short duration (4–30 d)	1.91	1.49	-1.016	4.843	0.200
	Long duration (> 30 d)	-1.91	1.69	-5.238	1.401	0.257
Parity	Primiparous	-				
	Multiparous	6.58	1.19	4.234	8.92	< 0.001
DIM (ln)		0.68	0.019	0.645	0.720	< 0.001
DIM		-0.07	0.001	-0.067	-0.066	< 0.001

### Calf ADG

The interaction between age and contact duration did not explain a considerable variation in ADG (*P* > 0.1 in univariable analysis). The final model for calf ADG consisted of the explanatory variables of contact duration, parity, calf sex, birth weight and age, in addition to the random effect of calf and herd. Age up to 195 d was used in the statistical analysis, as measurements were taken once a month for six months and observations > 195 d thus were scarce.

Overall, there was no effect of contact duration on ADG (*P* = 0.238, Table 5). Calves with NC, short duration and long duration of contact showed similar ADG for the first six months after calving (Table 5). Male calves had a tendency of gaining more weight than female calves (+ 0.08 ± 0.04 kg, *P* = 0.056, Table 5). The random effect of herd explained 1.6% of the residual variation and calf id 0.1%.

**Table 5:** Results from linear mixed model on calf ADG (kg/d)

		Coefficient	SE	95% lower CI	95% higher CI	*P*
Intercept		0.81	0.13	0.561	1.060	0.000
Contact duration	No contact (0–3 d)	-				
	Short duration (4–30 d)	0.08	0.05	-0.028	0.182	0.149
	Long duration (> 30 d)	0.08	0.05	-0.028	0.180	0.154
Parity	Primiparous	-				
	Multiparous	0.06	0.05	-0.028	0.147	0.183
Birth weight		-0.01	0.00	-0.006	0.004	0.542
Calf sex	Heifer	-				
	Bull	0.81	0.13	-0.002	0.154	0.056

## Discussion

The objective of this study was to gain a better understanding of how CCC impacts performance of cows and calves beyond separation, as previous research has yielded inconclusive results.

As hypothesized, daily machine milk yield was not affected by CCC for the period from separation to the end of lactation. The absence of differences in machine milk yield between both short and long contact duration groups, in comparison to the no contact group, is in contrast to some studies [17, 18], but in line with other studies [13, 14]. This lack of a difference may be explained by the interrelationship between frequent stimulation and udder emptying during early lactation. Milk production in cows follows a distinct pattern, starting at a low level after parturition and then rapidly increasing in early lactation due to high cell proliferation until reaching peak around 6–8 weeks [29]. Studies have demonstrated that more frequent or complete removal of milk from the mammary gland stimulates increased epithelial cell proliferation and acitivty, leading to enhanced milk production [30–32]. Therefore, increasing the frequency of udder emptying by calves has the potential to increase milk production [33]. However, it is uncertain whether calves sufficiently empty the udder at each suckling. Studies have shown that incomplete udder emptying can have a negative carry-over effect on subsequent milkings [34, 35]. The mammary gland is especially sensitive during early lactation, and milk remaining in the gland can inhibit further secretion through negative feedback mechanisms, leading to cell apoptosis and reduced activity with subsequent reduction in milk production [34]. Thus, actual milk production might be reduced in CCC cows already before separation [36]. However, before peak lactation there is a higher rate of cell proliferation than cell apoptosis. After peak lactation, there is a natural shift towards higher cell apoptosis than proliferation, accompanied by a steady decrease in milk production [37]. The majority of cows with short and long contact durations in the present study were separated from their calves before reaching peak lactation, thus there might still be sufficient time for cells to reach their secretory potential. Whereas if cows are separated from their calves after peak lactation, the mammary cells’ secretory potential might be lost, which could lead to a sustained negative effect the cows’ milk production.

It is however also worth noting that the herd contributing the most to the group with a short duration of contact was also the herd with the highest average annual milk yield.

The results indicate that CCC with different contact durations can be practiced without negative effects on machine milk yield after separation. Notably, sample size was low, and this conclusion only applies for the conditions at the three herds included. Contrary to what we hypothesized; our study revealed that ADG of calves was not positively affected by CCC, as both short and long contact duration groups showed comparable ADG as the no contact group for their first six months of life, supporting other studies [14]. Previous research has shown a strong association between calf ADG and milk allowance during the milk feeding period [38–40] and beyond weaning [39, 40]. Although we were unable to measure the amount of milk suckled by calves in our study, we gained valuable insights from the milk allowance provided by each herd for artificially reared calves. Notably, one of the herds implemented a relatively restrictive feeding regime, offering only 4–6 L/d of milk for artificially reared calves (*n* = 40). In contrast, artificially reared calves (*n* = 98) from the two other herds in our study received ad libitum milk, either partially or throughout the milk feeding period. Previous research has demonstrated a strong association between calf ADG and milk allowance during the milk-feeding period in artificial rearing [38–40]. One study compared ADG for different milk allowance and found that calves with high milk allowance (12 L/d) resulted in the highest ADG (0.88 kg/d) [41], which aligns closely with the ADG of NC calves in our study. Among CCC studies, one showed that artificially reared calves receiving high amounts of milk (12 L/d) even exhibited higher growth rates than suckling calves [21]. A recent CCC study also supported the strong association between calf ADG and milk allowance, as their control calves who were provided 12 L/d showed comparable ADG as the suckling calves [20]. Consequently, it is plausible that the generous milk allowance provided to NC calves in our study might account for the absence of ADG differences between contact durations.

Interestingly, we found no differences in ADG at any age, including the periods with suckling. However, one must consider that measurements were taken monthly, and many calves had a contact duration of less than a month. It is widely acknowledged that calves suffer from growth checks when both separated and weaned simultaneously [13, 20], and when separated but transitioned to artificial milk rearing during [42] or immediately after [43] separation. In the present study, one herd practiced abrupt separation from the dam and simultaneous weaning, while the other two herds practiced abrupt separation with a transition to artificial milk rearing. Training calves to suckle from an artificial teat after a period of natural suckling is known to be challenging [42, 44]. While it is unknown whether the farmers encountered this challenge, it is reasonable to assume that they may have faced difficulties. Given that all of the previously mentioned separation practices are known to be associated with weight checks, this suggests that many calves may have experienced a growth check already before the first monthly measurement, although not detected due to the study design. An improved ADG during the first month and a reduced ADG during the second month, depending on contact duration, might have been present but could have balanced each other out due to the limited data points during this initial two-month period. More frequent measurements during this critical phase would have offered a more comprehensive understanding of these early dynamics, which can potentially affect later growth potential. However, multiple previous studies have indeed identified such growth checks at separation in CCC systems [20, 21, 42], consequently the objective of the present study was not to delve into this aspect, but to compare growth beyond separation.

It is important to acknowledge the limitation of having farmers conducting the measurements, as it is possible that these measures were imprecise, making it difficult to observe any differences. Also, there could be potential variability in calf weight registrations between measurements. Maintaining the calf in the correct position and ensuring consistent tape tightness can be challenging, leading to possible discrepancies in weight measurements within and between observers (i.e., farmers). The limitations of these measures introduce a source of uncertainty that should be considered when interpreting the results of the study. While a live body weight of some calves to validate the farmers measures would have been ideal, it was deemed impossible under the given circumstances. The different way of CCC housing might also have influenced the data in our study, as the herd contributing with the majority of animals housed the cow with her calf in individual pens, compared to cows and calves from the other two herds being together with the herd within the freestall. Unfortunately, a notable number of herds had to be excluded from the source population, which limited the sample size of our study. The inclusion of more herds would have resulted in a larger sample size, enabling a better balance between exposure groups and more reliable detection of differences.

## Conclusions

In this unique prospective cohort CCC study, we could not detect any negative effects of CCC on machine milk yield after separation, nor any sustained effects on calf growth. Knowing from previous studies that there is a clear reduction in machine milk yield during nursing, this suggests that the saleable milk lost may not have been captured as a growth advantage by the calves in these herds. Future research should aim for a higher sample size and further explore possible differences between separation before and after peak lactation, knowing the underlying physiology behind milk synthesis and the present findings.

### Electronic supplementary material

Below is the link to the electronic supplementary material.

**Additional file 1**: Questionnaire in cow-calf contact prospective cohort study
A questionnaire used in a prospective cohort study comparing the effects of contact duration on cow and calf performance beyond separation within herds practicing both CCC and artificial calf rearing. The questionnaire was completed in collaboration with each farmer, and addressed herd data as herd size, the farmer’s method of CCC, milk allowance for artificially reared calves, herd size, and other relevant information

**Additional file 2**: Form for registrations in a cow-calf contact prospective cohort study
A form used in a prospective cohort study comparing the effects of contact duration on cow and calf performance beyond separation within herds practicing both CCC and artificial calf rearing. At calving, each farmer provided the required information (individual animal data as date and time of calving, cow and calf id, cow parity, any calving difficulties, calf sex, crossbreed of cow or calf (if any), whether the cow had prior experience caring for a calf, and the reason for why the specific contact duration was chosen for each pair), and registered monthly calf heart girth measurements

## Data Availability

The datasets collected and/or analysed during the current study are available from the corresponding author on reasonable request.

## Declarations

### Prior publication

Data have not been published previously.
