# Supplementary material for: Comparing the effects of contact duration on cow and calf performance beyond separation - a prospective cohort study
Source: Acta Vet Scand. 2024 May 22;66:21. doi: 10.1186/s13028-024-00741-1 (PMC11110400; doi:10.1186/s13028-024-00741-1)
Supplement: Supplementary file 1 — Additional file 1: Questionnaire in cow-calf contact prospective cohort study A questionnaire used in a prospective cohort study comparing the effects of contact duration on cow and calf performance beyond separation within herds practicing both CCC and artificial calf rearing. The questionnaire was completed in collaboration with each farmer, and addressed herd data as herd size, the farmer’s method of CCC, milk allowance for artificially reared calves, herd size, and other relevant information [file 13028_2024_741_MOESM1_ESM.pdf]

Additional file 1. PDF. Questionnaire in cow-calf contact prospective cohort study.

A questionnaire used in a prospective cohort study comparing the effects of contact duration on cow and calf performance beyond separation within herds practicing both CCC and artificial calf rearing. The questionnaire was completed in collaboration with each farmer, and addressed herd data as herd size, the farmer's method of CCC, milk allowance for artificially reared calves, herd size, and other relevant information.

General information about the herd

Herd name: ___________________________________

Producer number:___________________________________

County:______________________________

Tie-stall barn ☐ Free-stall barn ☐

Milking system

AMS DeLaval ☐ AMS Lely ☐ AMS SAC ☐ Parlor ☐ Pipeline ☐

Herd size: ______________

Average annual milk yield:_________________

1. General information

How long has the herd had a subpopulation with cow and calf together?___________________________

What prompted the herd to start keeping cow and calf together?

____

____

____

What benefits does the herd experience from keeping cow and calf together?

____

____

____

What are the challenges the herd experience by keeping cows and calves together?

____

____

____

How are cows and calves separated after the period together?

____

____

____

What are the criteria used to decide whether the pair should remain together or be separated after birth?

____

____

____

Are cows or calves in the herd vaccinated? No ☐ Yes ☐ If so, when and against what (e.g. Rotavec corona, Bovilis bovipast):________________________________

Is the colostrum quality measured?

- Always ☐
- Almost always ☐
- Sometimes ☐
- Never ☐

If colostrum quality is measured, what instrument is used?

- Colostrometer ☐
- Handheld refractometer ☐
- Digital refractometer ☐
- Other: _______________

What tools are used to record signs of estrus?

- Estrus calendar ☐
- Own notebook ☐
- Mobile ☐
- Do not record signs of estrus ☐
- Other: _______________

Environment and housing

Calving location:

- Calving pen ☐
  - Separate ☐
  - Group housing ☐
- Tie stall ☐
- Other:_____________________

Bedding in the calving area

Sawdust ☐

Straw ☐

Other: _______________

2. Subpopulation cow-calf

How are cows and calves kept together

In one pen alone ☐

Approximate size of the pen:______________________

In a larger pen/area with several cow-calf pairs ☐

Approximate size of the area:______________________

Freely in the loose housing ☐

Approximate size of the area:______________________

Other:____

How long are cows and calves kept together before they are separated? __________________

Can the calf nurse other cows? Yes ☐ No ☐

Colostrum

How is colostrum intake ensured?

- The cow and calf manage themselves, seldom help ☐
- The calf always receives colostrum from a bottle ☐
- The calf usually receives colostrum from a bottle only if it fails in nursing ☐
- The calf usually receives assistance to nurse only if it fails in nursing ☐

Milk allowance (during nursing period):

- Does the calf have free access to its dam milk (except during milking)? ☐
- Does the calf have free access to its dam only part of the day ☐ When:_________________________

Feed allowance (during nursing period)

In the calf creep, the calf has access to:

- Water ☐
- Silage ☐
- Hay ☐
- Concentrate ☐
- Does not have a creep: ☐

Other: ______________

From the dams' resources, the calf has access to:

- Water ☐
- Silage ☐
- Hay ☐
- Concentrate ☐
- Does not have access: ☐

Other: ______________

Do you observe that calves show interest for silage? Yes ☐ No ☐

From what age does the calf begin to show interest for:

**Silage?**		**Concentrate?**		**Hay?**	
0-2 weeks	☐	0-2 weeks	☐	0-2 weeks	☐
2-4 weeks	☐	2-4 weeks	☐	2-4 weeks	☐
6-4 weeks	☐	6-4 weeks	☐	6-4 weeks	☐
>6 weeks	☐	>6 weeks	☐	>6 weeks	☐

Weaning/separation

Is weaning and separation from the dam done simultaneously? Yes ☐ No ☐

The calf gets access to: Milk feeder ☐

Bucket ☐

Other:___________________

Cow and are separated:

Gradually ☐

Abruptly ☐

Cow and calf after separtion:

Can sniff each other Yes ☐ No ☐

Can see each other Yes ☐ No ☐

Can hear each other Yes ☐ No ☐

After separation, the calf is housed:

Individually ☐

In goup pen ☐

3. Subpopulation conventional rearing

When are cow and calf separated?

Immediately after calving ☐

0-60 min after calving ☐

>60 min after calving ☐

After the colostrum period ☐

Other:______________

During milk -feeding period, calves are housed:

- Individually ☐
- In group pen ☐
- First individaully for____d, the group pen ☐

Other:_______________

Colostrum

How is colostrum intake ensured?

- The cow and calf manage themselves, seldom help ☐
- The calf always receives colostrum from a bottle ☐
- The calf usually receives colostrum from a bottle only if it fails in nursing ☐
- The calf usually receives assistance to nurse only if it fails in nursing ☐

Milk allowance:

How is milk offered calves?

- Manually ☐
- Milk feeder ☐

Other:__________________

How often are calves offered milk?

**0-2 weeks of age:**		**2-4 weeks of age:**		**4-6 weeks of age:**		**>6 weeks of age:**	
2 times daily	☐	2 times daily	☐	2 times daily	☐	2 times daily	☐
3 times daily	☐	3 times daily	☐	3 times daily	☐	3 times daily	☐
>3 times daily	☐	>3 times daily	☐	>3 times daily	☐	>3 times daily	☐
Ad lib. access	☐	Ad lib. access	☐	Ad lib. access	☐	Ad lib. access	☐

Milk quantity and frequency is regulated by the feeder: ☐

Feeder settings:____

At each meal, calves are offered:

**0-2 weeks of age:**		**2-4 weeks of age:**		**4-6 weeks of age:**		**>6 weeks of age:**	
0-2L	☐	0-2L	☐	0-2L	☐	0-2L	☐
2-3L	☐	2-3L	☐	2-3L	☐	2-3L	☐
3-4L	☐	3-4L	☐	3-4L	☐	3-4L	☐
Ad lib.	☐	Ad lib.	☐	Ad lib.	☐	Ad lib.	☐

Milk quantity and frequency is regulated by the feeder: ☐

What type of milk is offered the calves?

Whole milk ☐
Milk replacer ☐
Soured milk ☐

Annet:_________________

Is milk with high somatic cell count given to calves? Yes ☐ No ☐ Sometimes ☐________________________________

Weaning/separation

At which age are the calves weaned? _______________ weeks

Is weaning and re-housing done at the same time? Yes ☐ No ☐

Feed allowance

During the milk-feeding period, calves have access to:

Individually housed:

Water ☐
Silage ☐
Hay ☐
Concentrate ☐
Other: ______________

In group pen:

Water ☐
Silage ☐
Hay ☐
Concentrate ☐
Other: ______________

After weaning, calves have access to:

Water ☐

Silage ☐

Hay ☐

Concentrate ☐

Other: _______________

Other notes:

____
____
____
____
____
____
____
____
____
