# Supplementary material for: Comparing the effects of contact duration on cow and calf performance beyond separation - a prospective cohort study
Source: Acta Vet Scand. 2024 May 22;66:21. doi: 10.1186/s13028-024-00741-1 (PMC11110400; doi:10.1186/s13028-024-00741-1)
Supplement: Supplementary file 2 — Additional file 2: Form for registrations in a cow-calf contact prospective cohort study A form used in a prospective cohort study comparing the effects of contact duration on cow and calf performance beyond separation within herds practicing both CCC and artificial calf rearing. At calving, each farmer provided the required information (individual animal data as date and time of calving, cow and calf id, cow parity, any calving difficulties, calf sex, crossbreed of cow or calf (if any), whether the cow had prior experience caring for a calf, and the reason for why the specific contact duration was chosen for each pair), and registered monthly calf heart girth measurements [file 13028_2024_741_MOESM2_ESM.pdf]

Additional file 2. PDF. Form for registrations in a cow-calf contact prospective cohort study.

A form used in a prospective cohort study comparing the effects of contact duration on cow and calf performance beyond separation within herds practicing both CCC and artificial calf rearing. At calving, each farmer provided the required information (individual animal data as date and time of calving, cow and calf id, cow parity, any calving difficulties, calf sex, crossbreed of cow or calf (if any), whether the cow had prior experience caring for a calf, and the reason for why the specific contact duration was chosen for each pair), and registered monthly calf heart girth measurements.

HERD NAME:

**GENERAL INFORMATION**	Calving (date + time)	Assistance during calving? (pulling, using a jack etc.)	Why did these become/did not become a CCC-pair?	Date of separation

COW ID:

Has the cow previously been taking care of a calf?		Non-veterinary health events (milk samplings, increased milking frequency, heat induction etc.)			
Yes	No	Date	Description	Date	Description
Is the cow a cross-breed?		Date	Description	Date	Description
Yes	No	Date	Description	Date	Description
If yes; what kind of cross-breed:		Date	Description	Date	Description

CALF ID:

Sex of calf		Chest width				Non-veterinary health events (electrolytes, charcoal paste, vitamins etc.)			
Bull Sale date (if applicable): _______________	Heifer	At birth	kg	Date	kg	Date	Description	Date	Description
Is the calf a cross-breed?		Date	kg	Date	kg	Date	Description	Date	Description
Ja	Nei	Date	kg	Date	kg	Date	Description	Date	Description
If yes; what kind of cross-breed:		Date	kg	Date	kg	Date	Description	Date	Description

Any questions? Contact Johanne Sørby: 95 11 67 17
